# Genetic structure of *Anopheles gambiae *populations on islands in northwestern Lake Victoria, Uganda

**DOI:** 10.1186/1475-2875-4-59

**Published:** 2005-12-09

**Authors:** Jonathan K Kayondo, Louis G Mukwaya, Aram Stump, Andrew P Michel, Mamadou B Coulibaly, Nora J Besansky, Frank H Collins

**Affiliations:** 1 Center for Tropical Disease Research and Training, University of Notre Dame, Notre Dame IN 46556-0369, USA; 2 Department of Entomology, UgandaVirus Research Institute (UVRI), Box 49 Entebbe, Uganda

## Abstract

**Background:**

Alternative means of malaria control are urgently needed. Evaluating the effectiveness of measures that involve genetic manipulation of vector populations will be facilitated by identifying small, genetically isolated vector populations. The study was designed to use variation in microsatellite markers to look at genetic structure across four Lake Victoria islands and two surrounding mainland populations and for evidence of any restriction to free gene flow.

**Methods:**

Four Islands (from 20–50 km apart) and two surrounding mainland populations (96 km apart) were studied. Samples of indoor resting adult mosquitoes, collected over two consecutive years, were genotyped at microsatellite *loci *distributed broadly throughout the genome and analysed for genetic structure, effective migration (Nem) and effective population size (Ne).

**Results:**

Ne estimates showed island populations to consist of smaller demes compared to the mainland ones. Most populations were significantly differentiated geographically, and from one year to the other. Average geographic pair-wise *F*ST ranged from 0.014–0.105 and several pairs of populations had Ne m < 3. The loci showed broad heterogeneity at capturing or estimating population differences.

**Conclusion:**

These island populations are significantly genetically differentiated. Differences reoccurred over the study period, between the two mainland populations and between each other. This appears to be the product of their separation by water, dynamics of small populations and local adaptation. With further characterisation these islands could become possible sites for applying measures evaluating effectiveness of control by genetic manipulation.

## Background

Malaria kills over a million people annually, most from sub-Saharan Africa [1]. Additionally, malaria mortality is on the rise, largely because of the emergence over the past two decades of widespread *Plasmodium *resistance to affordable antimalarial drugs [2]. Control approaches such as insecticide impregnated bed nets are also being challenged by the emergence of insecticide resistance in *Anopheles gambiae *and *Anopheles funestus*, the two primary malaria vectors in sub-Saharan Africa [3,4].

An alternative malaria control strategy being investigated in a number of laboratories is to genetically modify the vectorial capacity of vector populations by driving a genetic construct into the natural population. Genes that influence blood meal host selection, mosquito longevity, or *Plasmodium *survival have all been considered in genetic control, but most work has mainly focused on the identification of target genes that could modify the mosquito's ability to support *Plasmodium *sporogonic development [5-10]. The overall genetic control strategy depends not only on the identification and isolation of target genes but also on the development of effective transformation and drive systems and the development of potential field testing sites with vector populations that have been well characterized from the perspective of population biology and genetics. Although major advances are evident in genome resource development [11-13], target gene discoveries [14-18] and in genetic tool development [18,19], less progress has been made in characterizing vector populations in potential field trial sites.

Studies of population genetic structure are vital to any vector-targeted control measure, especially where *A. gambiae *is one of the vectors [20]. This species has a distribution that covers almost all of sub-Saharan Africa and genetic differentiation across populations of *A. gambiae *in Africa is complex. Microsatellite-, allozyme-and mitochondria-based studies have suggested extensive gene flow between populations in Senegal and western Kenya, a geographical distance of 6,000 km [21,22]. In contrast, analyses of frequencies of paracentric chromosomal inversions and ribosomal DNA markers have revealed high levels of population structure within sympatric populations of *A. gambiae *in West Africa [23-26]and high differentiation has been observed within Kenya across distances of 700 km traversing the Rift Valley [27]. In addition, *A. gambiae *island populations in Sao Tome [28] and *A. arabiensis *from the islands of Madagascar, Mauritius and Reunion have also shown extensive differentiation [29]. It is not clear if the lack of extensive differentiation among *A. gambiae *populations across wide geographical distances (Senegal and Kenya) is due to high rates of gene flow among large populations or shared ancestral polymorphisms from a recent population expansion event [30]. Physical barriers such as large areas of water and the Rift Valley are implicated in some instances where populations are highly differentiated, but chromosome inversion and molecular data also show clear evidence of pre-mating barriers producing reproductive isolation among sympatric populations [31,32].

This study is focused on population structure of *A. gambiae *on islands in Lake Victoria, a part of Africa where *A. gambiae *populations are generally thought to consist exclusively of the Savanna chromosomal form and the S molecular form. The purpose of this study was to use variation in microsatellite markers to investigate the genetic structure of populations of *A. gambiae s.s *on several islands in northern Lake Victoria with a view to determining whether geographic separation of these islands (from 20–50 km) was associated with any evidence suggesting restriction to gene flow. Chen and others [33] in a study very similar to this in objective, design and geographic area, looked at *A. gambiae *populations on islands 2.5–21 km apart in eastern Lake Victoria. Genetic structuring among the island populations and between islands and surrounding mainland populations was still detectable, though low. Isolated populations are potentially useful sites for studies to evaluate the potential impact of malaria control measures that involve genetic manipulation of natural vector populations.

## Methods

### Study sites and field collections

**Figure 1:**
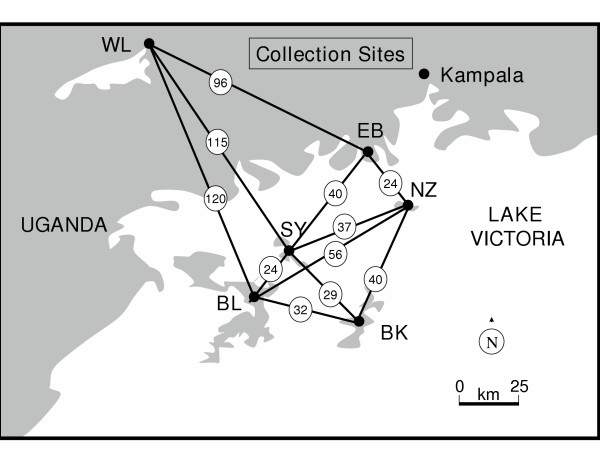
**Collection sites and surrounding Northern L. Victoria region, Uganda. **NZ = Nsadzi; BL = Bugala; SY = Sserinya; BK = Bukasa; WL = Wamala; EB = Entebbe. Separation distances (km) are circled.

The study area lies in Uganda, sub-Saharan Africa where malaria is endemic. Six *A. gambiae *populations involving one from each of four islands in northern half of Lake Victoria and two from the surrounding southern Uganda mainland were studied (Figure 1). The two inland populations consisted of Entebbe (EB), a peninsular jutting into Lake Victoria and Wamala (WL) located by the shores of a small inland lake 96 km away from Entebbe. The four islands are Nsadzi (NZ), Bugala (BL), Sserinya (SY) and Bukasa (BK). The islands are remote, but variable in size and ease of accessibility from the mainland and each other. Bugala, the largest, can be accessed from mainland by small boats and ferry whereas Nsadzi is the smallest and accessed only by boat. Bukasa lies farthest from the mainland sites. Apart from Entebbe, which is mainly residential, the rest of the locations are inhabited with people living by traditional farming subsisted with a little of fishing. Indoor resting adults from each population were captured at two or three separate villages by insecticide spraying done between 6 and 7 am. Populations were sampled as year (yr) 1 and as year 2 collections within a period of one to two years. Year 1 collections were made between November 2001 and February 2002. Year 2 collection, a replicate effort was performed between December 2002 and May 2003.

*A. gambiae *sensu lato (s.l.) of both sexes morphologically identified from other anophelines, based on a species identification key [34], were preserved in 80% alcohol and sent to the Center for Tropical Disease Research and Training-, University of Notre Dame (USA), for molecular identification and further analysis.

### Molecular species identification and marker genotyping

Genomic DNA was extracted from single mosquitoes by kit, using procedures in Mukwaya et al. [35], or, for yr 2, in 96 well-plates (Wizard SV-96 Genomic DNA Purification System, Promega) processed with a Biomek FX workstation (Beckman Coulter). Molecular species identification (PCR) was according to Scott *et al *[36]. Individuals that either did not amplify or gave incorrect sized product were excluded from subsequent genotyping analysis. This left 32 (Entebbe), 20 (Wamala), 33 (Bukasa), 32 (Sserinya), 36 (Bugala) and 36 (Nsadzi) individuals for the yr 1 sample set and 43 (Entebbe), 45 (Wamala), 47 (Bukasa), 20 (Sserinya), 92 (Bugala), 47 (Nsadzi) for yr 2. Yr 1 individuals were genotyped for variation at 17 microsatellite loci. Yr 2 replicate effort consisted of a 10 loci subset of the 17. Most loci used have been described elsewhere [21,37,38]. For those not previously described additional details are provided (see additional files 1, 2, 3).

Genotyping PCR was as follows: each 25 μl reaction contained 3.75 ng genomic DNA, 125 mM KCl, 25 mM Tris-HCl, PH 8.3, variable concentrations of MgCl2, 0.2 mM dNTP (Invitrogen, Carlsbad, CA) 0.011 mM each of either Fam, Tet and Hex or Blue, Green and Black Beckman coulter dye tagged forward primer and unlabeled reverse primer (Gibco/Brl, Gaithersburg, Md or Proligo LLC, Boulder, Co or Invitrogen) and 0.25 μl of home-made Taq DNA Polymerase. Yr 1 amplification was from GeneAmp 9600, whereas GeneAmp 9700 thermocycler (Applied Biosystems) was used for yr 2. The cycling program consisted of one cycle at 96°C, 5 minutes; thirty-five cycles of 94°C, 30 seconds; 55°C or optimal, 20 seconds; 72°C, 30 seconds; and one cycle of 72°C, 5 minutes. The Fam-Tet-Hex labeled PCR products constituted five of the 17 yr 1 loci set and were fragment size scored on the ABI 377 automatic sequencer using default settings of the genotyper software (Applied Biosystems). The remaining 12 loci of the data set were pool-plexed (two groups of six loci each) and genotyped using dye-labelled chemistry on the CEQ 8000 Beckman-Coulter capillary array genetic analysis system. Yr 2 were also pool-plexed into two groups (one of four and other of 6 loci) and similarly genotyped. A pool comprised; 1 μl product of each of 6 PCR reactions, 0.5 μl of a 400 bp size standard (Beckman-Coulter) and 30 μl SLS buffer (Beckman-Coulter). Both genotypers generate output fragment/allele sizes that are of within system reproducible non-integer lengths. Sizing of the outputs into integer length format useable by input files of the various genetic analysis programs is necessary. All Beckman-Coulter run samples were sized by binning, an automated process that relies on prior knowledge of the spectrum range of most possible apparent sizes for the generation of nominal fragment length sizes, with CEQ8000 software. This created an allele list that was used repeatedly to identify alleles whenever a locus was run under the same conditions. Sized alleles were manually inspected for correctness. Proper use of the binning option is described in the CEQ 8000 Genetic Analysis System User's Guide (Beckman-Coulter PN 608315).

### Data analysis

Within population deviations from Hardy-Weinberg (HW) expectations at each locus were tested by exact tests using an online (web) version of GENEPOP an update of version 1.2 [39] and also by ARLEQUIN [40]. Input files for both programs were conversions from the program Microsatellite Analyser (MSA) [41]. Conformity to Hardy-Weinberg expectations [H0 = of random union of gametes] was tested using the probability test. The possibility that heterozygosity deficiency may be the cause for departure from expectations was determined by setting the GENEPOP option [H1 = heterozygote deficiency]. To identify and correct genotyping errors in the data set the program MICRO-CHECKER [42] was used. Wherever presence of null alleles was suggested the data set adjustment procedure was accordingly applied to correct allele and genotype frequencies. The null-allele-adjusted data set was then used to explore the effect of null alleles on differentiation values resulting from the analysis. Linkage disequilibria, tests for independence between loci pairs, were done with web GENEPOP. Significance came from probability tests generated using Markov chain method at default parameter settings. Assessments of the six population deme sizes were achieved through estimations of effective population size (Ne) calculated from genetic data using the program MLNE [43]. The single isolated population option was used. Ne calculations by hand were performed to verify the MLNE results. Equations used in the hand calculations have been adequately described [28,44,45]. Essentially, current Ne, an estimate based on temporal variation in allele frequencies from one sampling time to another, was calculated across the ten shared yr 1 and yr 2 loci. The allele frequencies for both data sets were from MSA basic descriptive statistics outputs. The allele frequency change variance estimator Fc was chosen over Fa because it is less affected by the presence of an allele at time t but not time *0*, and over Fk for its superior Ne estimation when > 3 alleles per locus are present. Fc was calculated according to Nei and Tajima [46] and was weighted for multiple loci using equation (8) in Tajima and Nei [47];Waples [44] before substituting it into equation (11) in Waples [44] to get Ne. Twelve generations per year was adopted for t in equation 11 above. The presence of genetic differences across populations was determined from three measures of genetic variability; genic differentiation that tests for allelic distribution and genotypic differentiation for genotypic distribution; both done with GENEPOP. The third measure looked for variation in frequencies of observed heterozygosity among populations. This was done with the Friedman test from the statistical program package SPSS. The measures described only show the presence or absence of differences. For magnitude of differences or population structure three indices of differentiation were performed; multi-loci population pair-wise Wright's F-statistics (*F*ST); *R*ST [48] an index that differs from *F*ST mainly in assumption for model of microsatellite evolution; and Nm an index of migration rate. Pair-wise *F*ST were generated using MSA, *R*ST were got from the program ARLEQUIN [40] and Nem were estimated from formula; *F*ST = 1/1+4Nem adopted from equation 5.17 [49]. To further evaluate structure results, population pair-wise yr 1 and yr 2 *F*ST distributions were compared using paired t-test and the Wilcoxon-signed rank test from the SPSS package. Isolation by distance as the model explaining the observed population structure was tested by regression of Pair-wise Population *F*ST/(1 - *F*ST) against natural logarithm (ln) of pair wise geographical distances (Spearman Rank Correlation Test). The procedure was carried out online as computed in GENEPOP. Significance of the correlation coefficient was from Mantel tests. The geographical distances used were straight-line measurements between map points.

## Results

### Population composition, HW proportions and independence of loci

Molecular species identification [36] showed all samples that generated a PCR product, except some from Bukasa, to be *A. gambiae*. In Bukasa, all year one (yr 1) samples were *A. gambiae*, while the yr 2 collection was composed of about 80% *A. gambiae *and 20% *Anopheles arabiensis*. Within population Hardy-Weinberg (HW) equilibrium tests (Ho = random union of gametes, H1 = heterozygote deficit) found eight of 17 yr 1 loci in HW equilibrium across all populations. H544 was the only locus out of equilibrium in every population. The equilibrium status of the remaining 8 loci varied in a population dependent manner (see additional files 1, 2, 3). The Wamala population had the fewest loci departing from HW equilibrium, with only1 of the 17 with a heterozygote deficit. Bugala had the highest levels of departure from HW equilibrium, with six of 17 out of equilibrium. Yr 2 exhibited some deviations from equilibrium as well with significantly positive *F*is values in 17 of 60 tests. These HW deviations in both data sets indicated heterozygote deficiencies. MICRO-CHECKER, a program that statistically discerns out HW equilibrium errors resulting from null alleles from those by inbreeding or Wahlund effects based on distinctive allele class distribution signatures that each error carries [42], attributed all observed loci heterozygote deficiencies to null alleles. Linkage disequilibrium (LD) tests for loci pairings across the six populations were overall insignificant (*P *> *0.05*) except in three out of 136 (2%) pairings for yr 1. The three loci pairs that showed non-random association were H93 vs 29C1, H117 vs H544 and H117 vs MBP1B. All loci pairings used in yr 2 showed random association (LD tests *P *> *0.05*).

### Population genetic variability and differentiation

The loci were highly polymorphic in all populations as seen from number of alleles and heterozygosities (additional files 1, 2, 3). Although there were no significant across population differences in mean observed heterozygosities (Ho) in both years (Friedman test: χ^2 ^0.05,5,17 = *5.662*, *P *= *0.340 *for yr 1; yr 2 was similar), differences in allele composition and manner of pairing were evident from the highly significant genic and genotypic differentiation all *P *<<*0.0001*. Genic and genotypic differentiation tests are for allelic and genotypic distributions across populations, with the null hypothesis being (H0 = distribution identical across populations).

The effective population size (Ne), which is the size of an ideal population that behaves, with respect to allele fluctuations, like the observed real population, was estimated from the program MLNE. [43]. The Ne estimates showed differences in deme sizes between island and mainland populations (Table 1). The islands consisted of much smaller *A. gambiae *population sizes compared to mainland. Hand calculated Ne estimates (not shown) corroborated the MLNE values.

**Table 1:** Effective population size

*Ne estimates from temporal change in allele frequencies*		
Population	Ne	95% CI

NZ	397	123 - >9000
BL	403	140 - 4493
SY	234	78 - >9000
BK	677	161 - >9000
WL†	8,935	354 - ∞
EB†	8,810	338 - ∞

^† ^Denotes mainland populations.

### Degrees of genetic differentiation and population structure

Multilocus yr 1 *F*ST comparisons between population pairs revealed significant differentiation (Table 2). The across years population comparisons revealed substantial subdivision, except for the two mainland sites, in that comparisons of a particular location a certain year to itself another year were no lesser differentiated than those to different locations another year (Table 3). Likewise within yr 1 versus within yr 2 population pair comparisons comprised numerous instances of *F*ST variation in magnitudes (Table 4), even though statistically the yr 2 *F*ST distributions couldn't be shown to significantly differ from those of yr 1 (*P *= *0.119*, Wilcoxon signed ranks test). The yr 1 *F*ST distribution from a survey across the 10 loci used in year 2 (Table 4) was not significantly different from the distribution calculated using all 17 loci (t 0.05,14 = *0.05*, *p *= *0.961*, paired t-test). MICRO-CHECKER null allele adjusted data sets, when re-analysed for *F*ST gave similar levels of population differentiation as the unadjusted ones. Global *F*ST differentiation across combined all yr 1 loci among the four islands (*F*ST = 0.042, *P *<*0.001*) was comparable to that between island and mainland populations (*F*ST = 0.044, *P *<*0.001*) and only a little lower than was observed between the two mainland populations (*F*ST = 0.054, *P *<*0.001*) (see Table 5). The study loci were broadly spread across the genome and varied in their ability to capture inter population differences. Three adjacent study loci, MBP1A, MBP1B and 22C1, on the left arm of chromosome 2 starkly stood out from the others at capturing extreme population genetic differentiation values, all across except between island and mainland comparisons (Table 5). These three loci lie in the 2L*a *inversion at the proximal end and around its breakpoint neighborhood. When those three and H79 on 2R, the other inversion spanning locus, were excluded from the analysis, the between mainland population differences and the among islands differences substantially dropped leaving the between mainland and island and comparisons involving Bukasa as the remaining appreciable differentiations (Table 6). Moreover, H79, MBP1A, MBP1B and 22C1 alone account for nearly all the drop in *F*ST values observed when all null allele associated loci were excluded from the analysis (additional file 4).

**Table 2:** Differentiation among population pairs

	**NZ**	**BL**	**SY**	**BK**	**WL**†	**EB**†
**NZ**	-	0.030	0.001	0.015	0.074	0.096
**BL**	**0.014**	-	0.038	0.030	0.061	0.104
**SY**	**0.042**	**0.053**	-	0.015	0.099	0.105
**BK**	**0.038**	**0.070**	**0.033**	-	0.096	0.111
**WL**†	**0.048**	**0.047**	**0.079**	**0.080**	-	0.033
**EB**†	**0.078**	**0.105**	**0.067**	**0.057**	**0.054**	-

† Denotes mainland populations

Below diagonal in **bold**: *F*ST; above diagonal: *R*ST. Non significant pair wise indices (*P *> *0.05*) are underlined. Significance levels were got by permutation.

**Table 3:** One year to the other within population temporal, and among population geographic differentiations

	**NZ yr 2**	**BL yr 2**	**SY yr 2**	**BK yr 2**	**WL† yr 2**	**EB† yr 2**
**NZ yr 1**	**0.154**	0.051	0.095	0.048	0.031	0.067
**BL yr 1**	0.144	**0.048**	0.075	0.043	0.023	0.063
**SY yr 1**	0.107.	0.089	**0.083**	0.099	0.050	0.045
**BK yr 1**	0.100	0.097.	0.124	**0.095**	0.083	0.014
**WL**† yr 1	0.156	0.036.	0.088.	0.025	**0.011**	0.061
**EB**† yr 1	0.101	0.076	0.116	0.080	0.069	**0.007**

† Denotes mainland populations.

Global yr 1 Vsyr 2 FSTover all 6 loci: 0.057.

Global yr 2 Vsyr 1 FSTover all 6 loci: 0.093.

These six are those loci that shared a common genotyping protocol between both yrs.

Below diagonal: yr 2 Vs yr 1 sample population pair wise *F*ST; diagonal in **bold**: Intra population yr 1 against yr 2 sample *F*ST. Above diagonal: yr 1 Vs yr 2 sample population pair wise *F*ST. Non significant indices (*P *> *0.05*) are underlined. Significance levels were got by permutation.

**Table 4:** Comparisons between year 1 and year 2 pair-wise population *F*ST distributions

**Pair-wise Comparisons**	**Yr 1 *F*ST**	**Yr 1 *F*ST**	**Yr 2 *F*ST**
	**across 17 Loci**	**across 10 Loci**	**across 10 Loci**
NZ --------BL	0.014	0.01	0.092
.------------SY	0.042	0.035	0.046
.------------BK	0.038	0.039	0.145
.------------WL	0.048	0.065	0.103
.------------EB	0.078	0.077	0.078
BL---------SY	0.053	0.023	0.051
.------------BK	0.070	0.052	0.044
.------------WL	0.047	0.056	0.069
.------------EB	0.105	0.080	0.055
SY---------BK	0.033	0.032	0.107
.------------WL	0.079	0.087	0.045
.------------EB	0.067	0.088	0.093
BK---------WL	0.08	0.093	0.077
.------------EB	0.057	0.063	0.072
WL---------EB	0.054	0.033	0.068

Note. The 10 are part of the initial 17 loci.

**Table 5:** Locus specific *F*ST group comparisons

Locus	Among four island Populations	Between two mainland populations	Between Island and Mainland	Among all populations
**ID1**	0.002 ns	0.008 ns	0.018*	0.009 ns
H99	0.072***	0.085***	0.029**	0.075***
H53	0.005 ns	0.006 ns	0.004 ns	0.002 ns
H145C/D	0.032**	0.021 ns	0.067***	0.056***
22C1	0.112***	0.138***	0.003 ns	0.100***
MBP1A	0.205***	0.323***	0.034**	0.211***
MBP1B	0.062***	0.126***	0.003 ns	0.064***
H117	0.002 ns	0.008 ns	0.126***	0.069***
H197	0.004 ns	0.008 ns	0.015**	0.008 ns
H79	0.005 ns	0.025*	0.015*	0.015*
H577	0.003 ns	0.013 ns	0.003 ns	0.000 ns
H544	0.080***	0.054*	0.032**	0.077***
H817	0.009 ns	0.039*	0.118***	0.074***
H93	0.002 ns	0.004 ns	0.011*	0.007*
**29C1**	0.004 ns	0.013 ns	0.021*	0.010 ns
H158	0.001 ns	0.007 ns	0.011*	0.004 ns
**33C1**	0.097***	0.010 ns	0.22***	0.172***
Overall	0.042***	0.054***	0.044***	0.057***

*P <*0.05*, **P <*0.01*, ***P <*0.001*. ns = not significant.

Tri-nucleotide loci are shown in **bold**, the rest are di-nucleotide repeats.

**Table 6:** Yr 1 population *F*ST differentiations excluding, MBP1A, MBP1B, 22C1 and H79, the loci in neighbourhood of known inversions

	NZ	BL	SY	BK	WL†
BL	0.005	-			
SY	0.014	0.007	-		
					
BK	0.019	0.047	0.035		
WL†	0.055	0.049	0.057	0.072	
EB†	0.056	0.069	0.080	0.060	0.018

^† ^Denotes mainland populations.

Non significant pair wise values are underlined.

**Figure 2:**
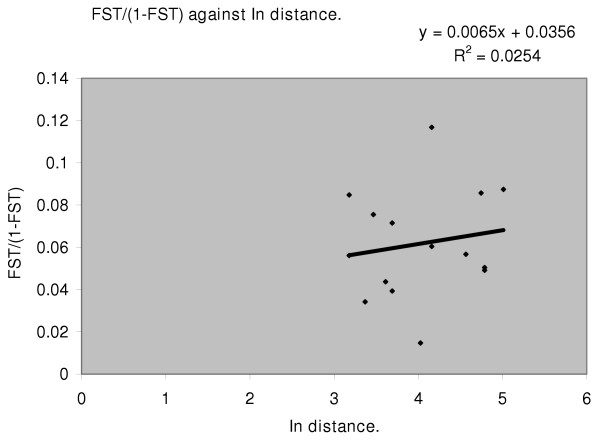
**The effect of distance on population differentiation. **The regression was made using *F*ST/(1 - *F*ST) against natural log (ln) separation distance. The equation describes best-fit regression line and shows little correlation between geographic location and degree of differentiation or genotype count.

Estimates across all the 17 yr 1 loci of the effective migration (Nem) showed the existence of structuring with varying degrees of restriction to gene flow between population pairs (Table 7). Geographical distance as the main factor explaining differentiation patterns was found to be insufficient. The observed population structure was not compatible with the isolation by distance model when regression between *F*ST/(1 - *F*ST) versus ln distance was evaluated (Mantle test; *P *= *0.787*), in that there was little correlation between geographical distance and degree of differentiation (Fig 2).

**Table 7:** Levels of gene flow

	Effective migrants per generation (Nem) between populations estimated from *F*ST				
Population	NZ	BL	SY	BK	WL†
BL	16.99				
SY	5.72	4.46			
BK	6.36	3.31	7.30		
WL†	4.96	5.09	2.92	2.86	
EB†	2.95	2.14	3.50	4.14	4.41

^† ^Denotes mainland populations Gene flow estimated using all 17 yr1 loci.

## Discussion

The studied samples consisted of indoor resting, insecticide spray-catch specimens. Although there have been occasional indicators from other studies of *A. gambiae *that certain genotypes are associated with different resting behaviors  [50-52], overall the *A. gambiae *populations in East Africa are panmictic, even taking into account different resting behaviors [53]. So it can be taken that indoor sampling was adequately representative.

**Figure 3:**
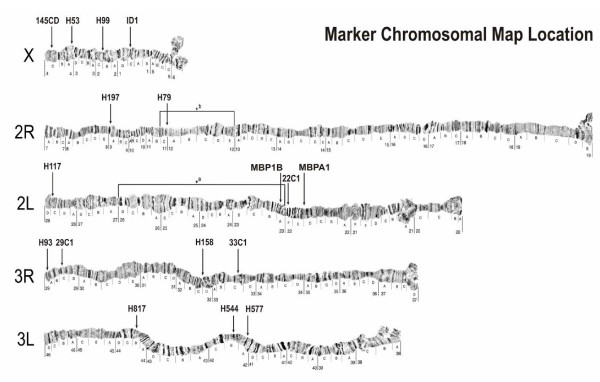
**Marker and inversion chromosomal map locations. **Study microsatellite marker map positions alongside known inversions found in East African *Anopheles gambiae *populations.

Neutrality from selection and genetic independence of loci used in genetic studies are required prior to analysing genetic variation at multiple microsatellite loci for population structure. Three pairings involving 5 loci in this study showed nonrandom association. All loci used in the study have known chromosome map locations (Figure 3). It is likely that H93 and 29C1 are unlinked because they are one chromosomal subdivision apart and located at the telomeric end, a region of chromosome where recombination is less restricted. However, in the islands population study by Chen *et al *[33] linkage disequilibria was also found among some of their loci pairs so it is plausible H93 and 29C1 linkage disequilibria could be quite incomplete through hitch-hiking to a nearby gene under selection. There is no direct genetic evidence to support this though. H117 and MBP1B although situated on the same chromosome arm, the two loci are far apart and sit in different chromosomal environments. H117 sits on telomeric end whereas MBP1B is located in an inversion and for standard arrangement more than six divisions upstream (Figure 3). Therefore, little possibility for linkage is expected, be it in the standard or inverted arrangement. H117 and H544, the last of non-freely associating pairs, map to different chromosomes and hence are not in the same linkage group so they are more likely to be unlinked. Finally, three instances of significance out of 136 tests (~2%), as is the case for this data set, are not above the range expected by chance alone at α = 0.05.

Deviations from HW were registered at certain loci. Deviation from frequencies expected from HW is not uncommon and while a potential indicator for selection at a locus [54] it is considered unlikely in most of the loci, as majority of them (15 of the 17, see additional file 1) have previously been used without evidence of selection. Moreover, departure from HW can arise from a variety of other causes including presence of null alleles [55,56], hidden sub-structure and inbreeding in a population [57]. These collections were made from more than one village so patchy distribution within each population could, if present, affect the equilibrium. Little is actually known about breeding behavior, deme sizes and distribution in these populations. Although some slight inbreeding has recently been suggested for natural *A. gambiae *populations in East Africa [58], which if present could account for the deviations, the expected associated inbreeding signature of genome-wide departures from HW equilibrium was not found. Inbreeding in these samples being the cause of non-equilibrium was ruled out due to lack of such genome-wide departures from HW equilibrium in any of the populations. The observed HW equilibrium departures were locus specific. Moreover, earlier studies on other East African populations found random mating [21,51]. The HW deviations were attributed to null alleles by the MICRO-CHECKER program. This program statistically discerns out HW equilibrium errors resulting from null alleles from those by inbreeding or Wahlund effects as each carries a distinct allele class distribution signature. In fact such, locus-specific, HW deviation patterns resulting from null alleles have been previously encountered by other investigators. Donnelly *et al *[57] found null alleles responsible for 5 of 6 loci HW deviations while studying structure in *A. arabiensis*, whereas Lehmann *et al *[27] had 4 of 9 loci showing some instances of non-equilibrium in their *A. gambiae *study. The impact of null alleles in the data on the analysis was negligible based on the fact that re-analysis of adjusted data sets returned similar differentiation values.

Populations, other than the mainland ones, were significantly differentiated across the years to the extent that they were substantially different even from themselves, from one year to another. This is evidence, in these populations, for demographic instability probably emanating from seasonal changes and is indicative of small population sizes on the islands. In spite of overall differentiation across the years; the within yr 1 *F*ST distribution when arrayed against the within yr 2 *F*ST distribution did not statistically significantly differ according to the Wilcoxon-signed ranks test perhaps because of some population pair differences that were exactly recaptured a year later. The yr 1 vs yr 2 irregularity of appearance of *A. arabiensis*, in Bukasa samples is probably a sampling-time artifact that probably caught them out of synch. Yr 1 one samples were collected in the months of November through February, a period that falls in the dry season, whereas the yr 2 collections spanned through a dry and wet season (see methods). Bukasa yr 1 samples were collected November/December 2001, while yr 2 got collected during months of April/May 2003. These samples, while spatially true replicates, were not replicates temporally. Relative frequencies of various members of the *A. gambiae *complex are known to fluctuate with season and geographical location [23].

Effective population size (Ne) comparisons across populations are usually not factored into structure analysis due to lack of reliable direct methods of estimates [27,44]. The study generated indirect Ne estimates show that the islands on the whole have lower deme sizes compared to the mainland. The island Ne's were in the hundreds, whereas mainland effective populations sizes were in the thousands, a result that is consistent with the conclusion arrived at earlier that small population sizes exist on these islands. In contrast, the western Kenya island study [33] inferred a large effective population size, in both, the islands and mainland, based on their comparable degrees of polymorphism in terms of average number of alleles and levels of observed heterozygosity. However, Nes inferred that way are only qualitative and do not take into account actual allele constitution or make up the way changes in individual allele frequencies in the method of Ne calculation used this study does. Therefore, the present study's Nes because of their being quantitative are more exact. A previous study on *A. gambiae *population size in Kenya [45] corroborates the large mainland Ne estimates.

Within population genetic diversity was high both on the islands and the mainland considering heterozygosity levels and the number of alleles seen (additional files 1, 2, 3). Across population differentiation, with respect to allele frequencies and genotype constitution, was high in all cases. The level of genetic differentiation among islands and mainland populations was considerable according to multi-loci pair-wise *F*ST (Table 2). *F*ST and *R*ST both estimate the amount of differentiation but each suits different scenarios. *F*ST assumes infinite allele mutational (IAM) model while *R*ST assumes and requires strict adherence to a step-wise mutation (SMM) model for microsatellite evolution [48]. Of the repeat motif classes in the marker sets used only the tri-nucleotide (3 bp) repeat loci satisfactorily conformed to the SMM with regard to generating products consistent with a series predictable from the repeat motif inside a constant flanking sequence; because several alleles among the dinucleotide loci appeared to be separated by only one nucleotide which leads to inconsistencies and mis-scoring. Therefore, *F*ST values were regarded as the more robust ones. Low but significant genetic structure was found among the island population (*F*ST = 0.019) and between island and mainland populations (*F*ST = 0.003) situated from 3–20 km apart in the Western Kenya-Lake Victoria study [33]. These Ugandan island populations situated 20–50 km apart are more differentiated (Table 5) than those in the Western Kenya Lake Victoria island study, perhaps due to the longer separation distances involved. Across 17 loci, the observed levels of differentiation among the island populations did not much differ from those seen between islands to mainland or between the two mainland populations. However, this effect was not identical genome-wide in that all loci did not capture it to the same extent. They greatly varied in their ability to capture inter-population differences. Among those loci that captured significant group differences (Table 5) it is apparent that each had its own independent differentiation rate across the populations. The loci in the inversions particularly the three involved with 2L*a *extremely differentiate the populations. Excluding them from the analysis substantially drops most inter island differences and the inter mainland pair difference although island to mainland difference and Bukasa differences are less affected (Table 6). Although the effect of inversions on gene flow in *A. gambiae *is unknown the above result points to possible role of inversion situated loci in driving population differentiation. In fact, 2L*a *and some 3R inversions have shown clines with aridity [23,59,60] and association with particular resting behaviors[51,61], such that genes within them are probably involved in environmental adaptations. Site ecological differences are evident across these populations. The islands are mostly forested and covered in rush green natural vegetation. The inland Lake Wamala population lies in a lesser naturally-vegetated, drier, wooded grassland-like region with farm crops. The mainland Entebbe area is somewhat intermediate; a peninsular extending from a forested mainland on one end and becoming less vegetated heading towards the lake. While it is possible in light of the above that some of the observed variation between the populations is shaped by differential adaptation and small population size effects, the rest, at mutation equilibrium, is then accounted for by restrictions to gene flow. This gene flow restriction is not likely to arise from chromosomal form diversity because populations in this region are thought to consist of only the savanna form. It is possibly arising from barriers to dispersal.

The indices of effective migration (Nem) indicate that gene flow is indeed substantially though not completely restricted, between many pairs (Table 7). It is strongly evident that the nature of the barrier responsible for the observed population structure has less to do with sheer geographical distance (Figure 2), than with water separation: Entebbe peninsular is geographically farther from the inland Wamala population than from any island population, however, it is less isolated genetically from Wamala than from any of the islands. The distances separating these populations (see Figure 1) are beyond both the normal 1 km *A. gambiae *flight range [62] and 7 km wind assisted flight range [34]. While it is not absolutely inconceivable that wind could be a factor in this, mosquito dispersal between these populations is more likely to be man assisted. However, conclusions about effective migration levels derived from Nem values should be interpreted with care for several reasons: Foremost, Nem were indirectly estimated from *F*ST. The relationship between Nem and *F*ST is non linear so any errors in *F*ST are magnified in Nem. Secondly, although an *F*ST gives a measure of relative amount of differentiation between a population pair it is still confounded by time in sense that the derived Nem is based on structure that has been generated over many generations so cannot distinguish recurrent from ancestral gene flow. Actually it is advised that all indirectly calculated migration rates be viewed cautiously [63]. This study had scope to primarily study differentiation and not to measure present active migrations or actual dispersals (Nm) between populations and so the Nem are only portrayals of gene flow rates in terms of effective migration in light of the observed levels of differentiation. To get actual dispersal or migration levels would require use of direct methods of acquisition such as capture-recapture. The cost of these direct methods has become affordable in recent years [63].

It was found that these island populations in North Western Lake Victoria region are substantially differentiated from the mainland and some of each other. It also is that this differentiation is strongly shaped by physical barriers to dispersal or gene flow, processes associated with small population sizes and possibly also by ecological adaptation because the levels of differentiation found contrast starkly with what has mostly been reported for *A. gambiae *populations around the continent. Most of the *F*ST were much higher than (*F*ST = 0.014) expected for mainland populations at similar range of separation distances [64-66]. The differentiations in several instances were more like those seen across the Rift Valley (mean *F*ST = 0.104, [27]); island populations in Sao Tome [28] and *A. arabiensis *amongst Madagascar, Mauritius and Reunion (*F*ST 0.08 - 0.215, [29]) that involved barriers to gene flow. Although not in complete genetic isolation since only gene flow from Nem levels of 2 and less could allow this [49], they are some of the most differentiated *A. gambiae *populations among those studied to date. This high differentiation and smaller population size confers to them some practical importance in fight against malaria because completely, or in their absence, even nearly isolated small vector populations could be used as field sites for evaluating impact of malaria control measures including those using genetic manipulations. However, before they are adopted for this role extensive additional studies must be carried out. There is need to establish for example, what the exact nature of the barrier is. Is it just water or is there more to it like some other yet unknown physical aspect? In this way, potential ways of its compromise could be monitored during duration of trials. It would be interesting to figure out the origin of the observed differentiation. It makes a huge difference to understand whether this is recurrent or historical gene flow. Among the recurrent processes involved it is crucial to know the relative significance of the factors at play. For instance, is the differentiation primarily driven by extinctions on islands followed by re-colonization from elsewhere or just drift fluctuation followed by recovery from extensive births without significant immigrants impacts. These pertinent studies could be done with use of markers that have lower mutation rates to microsatellites and are able to look farther back into the past and incorporating the findings with those from direct measures of present day migration rates. It is still intriguing that there is substantial differentiation amongst these populations in spite of possible passive mosquito dispersal (by human activity) across the barrier through ferry or boat traffic (Fig. 1). This could mean that passive dispersal, though commonly implicated, might not be as effective as widely thought. The role and extent of passive mosquito dispersal in natural conditions need to be empirically determined.

## Conclusion

These lake islands are significantly genetically differentiated from the two mainland populations. Several of them are also differentiated from one another. The genetic differences are real for they reappeared in yr 2. These genetic differentiations are possibly the product of several factors: the islands physical separation across water, effects of their small population size and local ecological adaptation. Although the relative contribution of each differentiating factor is yet to be quantified, when done these islands could become candidate sites for measures evaluating effectiveness of control by genetic manipulation. Lastly, this study adds to the body of data that has found substantial structure among *A. gambiae *populations across physical barriers.

## Authors' contributions

JKK carried out study design, sample processing, data acquisition, analysis and interpretation and manuscript preparation. LGM conceived of the study. AS substantially participated in data analysis. APM was key in data collection techniques and analysis. MBC greatly helped draft the manuscript. NJB helped with marker selection resources. FHC participated in the design of the study and substantially helped draft the manuscript.

## Supplementary Material

Additional File 1 **Details and variability summary of 17 yr 1 microsatellites**. A table of yr 1 sample size, loci names and their chromosomal location, repeat motif, number of alleles, heterozygosities and population breeding coefficients. Click here for file

Additional File 2 **Details and variability summary of 17 yr 1 microsatellites continuation**. A continuation table of yr 1 sample details from additional file one. Click here for file

Additional File 3 **Details and variability summary of 17 yr 1 microsatellites continued**. Caption for the contents in additional files 1, and 2. Click here for file

Additional File 4 **Yr 1 population *F*ST differentiations excluding loci that showed evidence of null alleles**. This is an analysis of year 1 differentiations with all loci that showed evidence of null alleles excluded. Click here for file
