# Supplementary material for: Genetic structure of Anopheles gambiae populations on islands in northwestern Lake Victoria, Uganda
Source: Malar J. 2005 Dec 9;4:59. doi: 10.1186/1475-2875-4-59 (PMC1327676; doi:10.1186/1475-2875-4-59)
Supplement: Additional File 1 — Details and variability summary of 17 yr 1 microsatellites. A table of yr 1 sample size, loci names and their chromosomal location, repeat motif, number of alleles, heterozygosities and population breeding coefficients. [file 1475-2875-4-59-S1.pdf]

Additional file 1- Details and variability summary of 17 yr 1 microsatellites

**Locus**		**NZD (N= 36)**	**BLA (N= 36)**	**SYA (N= 32)**	**BKA (N=33)**	**WLA (N= 20)**	**EBB (N= 32)**	**Citation or primer sequence Fwd/ Rev**
ID1	Alle	4	4	4	5	2	5	Lehmann et al [21]
X : 1D	Ho	0.5	0.42	0.52	0.47	0.5	0.59	
[CCA]$_5$	He	0.43	0.43	0.53	0.46	0.49	0.6	
	Fis	-0.16	0.03	0.01	-0.02	-0.03	-0.01	
H99	Alle	6	6	4	6	4	6	Wang et al [37].
X : 2C	Ho	0.64	0.58	0.5	0.69	0.8	0.72	
[GT]$_8$	He	0.73	0.77	0.59	0.79	0.76	0.76	
	Fis	0.12	0.23	0.14	0.13	-0.07	0.04	
H53	Alle	6	6	6	6	7	7	Wang et al [37]
X : 4A	Ho	0.71	0.72	0.63	0.81	0.8	0.74	
[GT]$_7$	He	0.79	0.78	0.77	0.75	0.81	0.77	
	Fis	0.09	0.06	0.17	0.1	0.01	0.03	
H145C/D	Alle	4	4	4	4	5	6	Wang et al [37]
X : 4C	Ho	0.61	0.51	0.59	0.52	0.53	0.59	
[GT]$_{11}$	He	0.74	0.73	0.72	0.63	0.72	0.79	
	Fis	0.17	**0.29**	0.17	0.17	0.26	**0.24**	
22C1	Alle	5	6	5	4	5	6	Wang et al [37]
2L : 22F	Ho	0.53	0.66	0.56	0.72	0.7	0.68	
[TG]$_7$	He	0.69	0.69	0.72	0.66	0.64	0.72	
	Fis	**0.23**	0.04	**0.22**	0.11	0.11	0.05	
MBP1A	Alle	7	7	8	7	4	8	atgcgatgaatcttggcttc / ggaatttcgggacaagatga
2L : 23A	Ho	0.58	0.57	0.56	0.38	0.7	0.53	
[CA]$_6$	He	0.7	0.53	0.59	0.7	0.62	0.53	
	Fis	0.17	-0.08	0.04	**0.46**	-0.15	-0.01	
MBP1B	Alle	10	9	10	12	8	8	ggcaggaaaggttttggaat / catcagcaacgctagtggaa
2L : 23A	Ho	0.36	0.54	0.5	0.37	0.67	0.47	
[TG]$_{11}$	He	0.86	0.77	0.89	0.89	0.83	0.77	
	Fis	**0.58**	**0.29**	**0.43**	**0.58**	0.19	**0.39**	
H117	Alle	5	5	5	5	7	5	Wang et al [37]
2L : 28C	Ho	0.31	0.43	0.69	0.7	0.65	0.63	
[GT]$_{6+4}$	He	0.74	0.68	0.73	0.74	0.84	0.76	
	Fis	**0.58**	**0.37**	0.05	0.05	0.21	0.17	
