# Supplementary material for: Genetic structure of Anopheles gambiae populations on islands in northwestern Lake Victoria, Uganda
Source: Malar J. 2005 Dec 9;4:59. doi: 10.1186/1475-2875-4-59 (PMC1327676; doi:10.1186/1475-2875-4-59)
Supplement: Additional File 2 — Details and variability summary of 17 yr 1 microsatellites continuation. A continuation table of yr 1 sample details from additional file one. [file 1475-2875-4-59-S2.pdf]

Additional file 2- Details and variability summary of 17 yr 1 microsatellites continuation

H197	Alle	10	10	9	8	8	8	Wang et al [37]
2R : 9A	Ho	0.8	0.79	0.77	0.85	0.65	0.6	
[GT]$_{8}$	He	0.84	0.88	0.79	0.79	0.78	0.82	
	Fis	0.04	0.09	0.02	-0.08	0.15	0.27	
H79	Alle	4	4	5	5	4	4	Zheng et al [38]
2R : 11C	Ho	0.69	0.71	0.58	0.67	0.6	0.71	
[GT]$_{20}$	He	0.7	0.7	0.73	0.66	0.61	0.68	
	Fis	0.01	-0.04	**0.2**	-0.02	0.01	-0.06	
H577	Alle	8	8	7	7	5	8	Zheng et al [38]
3L : 42A	Ho	0.57	0.61	0.68	0.64	0.7	0.65	
[GT]$_{16}$	He	0.6	0.7	0.74	0.69	0.72	0.69	
	Fis	0.03	0.12	0.08	0.06	0.02	0.06	
H544	Alle	4	5	6	7	5	7	Wang et al [37]
3L : 42B	Ho	0.22	0.22	0.2	0.33	0.3	0.41	
[GT]$_{7}$	He	0.73	0.65	0.71	0.78	0.74	0.76	
	Fis	**0.69**	**0.66**	**0.72**	**0.57**	**0.59**	**0.46**	
H817	Alle	5	6	6	5	6	6	Zheng et al [38]
3L : 44B	Ho	0.47	0.62	0.75	0.61	0.6	0.66	
[GT]$_{8}$	He	0.58	0.72	0.66	0.66	0.65	0.75	
	Fis	0.19	0.13	-0.14	0.07	0.06	0.11	
H93	Alle	9	12	8	12	7	10	Zheng et al [38]
3R : 29A	Ho	0.83	0.64	0.63	0.76	0.8	0.71	
[GT]$_{4+7}$	He	0.82	0.87	0.85	0.86	0.78	0.86	
	Fis	-0.03	**0.26**	**0.26**	0.11	-0.04	0.17	
29C1	Alle	2	4	4	4	2	2	Lehmann et al [21]
3R : 29C	Ho	0.46	0.51	0.43	0.42	0.35	0.55	
[TGA]$_{11}$	He	0.5	0.52	0.56	0.55	0.36	0.46	
	Fis	0.07	0.01	**0.23**	**0.22**	0.01	-0.22	
H158	Alle	8	8	8	8	7	8	Wang et al [37]
3R : 32D	Ho	0.64	0.75	0.72	0.67	0.75	0.66	
[GT]$_{12}$	He	0.64	0.72	0.66	0.68	0.78	0.75	
	Fis	-0.01	-0.06	-0.1	0.01	0.03	0.12	
33C1	Alle	5	4	3	3	5	5	Lehmann et al [21]
3R : 33C	Ho	0.58	0.31	0.31	0.3	0.55	0.48	
[AGC]$_{6}$	He	0.68	0.47	0.39	0.56	0.76	0.66	
	Fis	0.13	**0.33**	0.19	**0.45**	0.27	**0.26**	
