# Supplementary material for: Genetic structure of Anopheles gambiae populations on islands in northwestern Lake Victoria, Uganda
Source: Malar J. 2005 Dec 9;4:59. doi: 10.1186/1475-2875-4-59 (PMC1327676; doi:10.1186/1475-2875-4-59)
Supplement: Additional File 3 — Details and variability summary of 17 yr 1 microsatellites continued. Caption for the contents in additional files 1, and 2. [file 1475-2875-4-59-S3.pdf]

Additional file 3- Details and variability summary of 17 yr 1 microsatellites continued.

Loci name, chromosomal arm subdivision and basic repeat unit are provided. Positions and primer specifics of previously used markers determined as cited. Positions of the previously unstudied got from sequence BLASTS against the *Anopheles gambiae* genome. N denotes sample size, All: number of alleles seen at the locus, H_o: Observed heterozygosity, H_e:expected heterozygosity, F_{is}: inbreeding coefficient. Significant F_{is} values ($P < 0.05$ after Bonferroni correction) are given in **bold.** Underlined are the 10 loci of the random subset used for the replicate Yr 2 effort.
