# Supplementary material for: Genetic structure of Anopheles gambiae populations on islands in northwestern Lake Victoria, Uganda
Source: Malar J. 2005 Dec 9;4:59. doi: 10.1186/1475-2875-4-59 (PMC1327676; doi:10.1186/1475-2875-4-59)
Supplement: Additional File 4 — Yr 1 population FST differentiations excluding loci that showed evidence of null alleles. This is an analysis of year 1 differentiations with all loci that showed evidence of null alleles excluded. [file 1475-2875-4-59-S4.pdf]

Additional file 4- Yr1 population F_{ST} differentiations excluding loci that showed evidence of null alleles

[†] Denotes mainland populations
Non significant pair wise values are underlined.

	NZ	BL	SY	BK	WL[†]
BL	0.000	-			
SY	0.018	0.008	-		
BK	0.013	0.015	0.021	-	
WL[†]	0.046	0.024	0.041	0.038	-
EB[†]	0.040	0.034	0.051	0.022	0.016
